# Biophysics of Bacterial Colonial Structures and the Occupancy of Microecological Spaces

**DOI:** 10.3390/biology15010056

**Published:** 2025-12-28

**Authors:** Fernando Baquero, Teresa M. Coque, Natalia Bastón-Paz, Ana Elena Pérez-Cobas

**Affiliations:** 1 Department of Microbiology, Ramón y Cajal Institute for Health Research (IRYCIS), Ramón y Cajal University Hospital, 28034 Madrid, Spain; teresacoque@gmail.com (T.M.C.); natalia.baston.paz@gmail.com (N.B.-P.); anaelena84@gmail.com (A.E.P.-C.); 2 CIBER in Epidemiology and Public Health (CIBERESP), 46980 Madrid, Spain; 3 CIBER in Infectious Diseases (CIBERINFEC), 28029 Madrid, Spain

**Keywords:** microbial biophysics, colonies, biofilms, colonial organisms, microbial masses, multicellularity, microecology, microbiota, corrosion

## Abstract

The process of bacterial reproduction on surfaces conducive to growth forms colonies, which are defined as physical bodies with functional and environmental effects. This phenomenon can be conceptualized as transforming biological processes into physical phenomena. Large bacterial multicellular aggregates can be conceptualized as physical entities, produced by “colonial organisms”, thereby transforming physics into biology. The formation of colonies requires surfaces, typically hydrogels or liquid–air interfaces, but also hard solid surfaces. Bacterial cell layers also contribute to the production of surfaces. Within a typical 3D-shaped, frequently domed colony, a variety of microcompartments form at the intersections of gradients that diffuse from its aerial and surface limits, leading to cellular functional diversity. This heterogeneity can lead to physical changes and fractures in the colony material, leading to the formation of fluid microchannels. The second primary type of colony is the 2D-shaped form that spreads over larger surfaces and is known as a biofilm. These physical structures possess significant water content, which is retained by a bacterial-excreted exopolymer. Biofilms are structurally organized as multilayer structures that can expand in the space through the lateral slippage of a more fluid overlayer on top of the surface-attached layer. The dissemination of biofilms may entail the integration of additional bacterial colonies, thereby giving rise to complex biofilms. The physical occupancy of microenvironments by colonies created on surfaces of higher organisms or on environmental surfaces exerts a significant influence on fluid mechanics and the functioning of organisms and ecosystems. In addition, colonies also contribute to the pathology of industrial constructions and devices, often leading to microbiologically influenced electrochemical corrosion, which results in material degradation.

## Simple Summary

Bacterial colonies constitute physical entities. Thus, beyond their biological activities, microorganisms exert effects arising from their physical occupation of microecological spaces, particularly altering the flow of fluids within macroorganisms and the environment.

## 1. Introduction: Colonial Organisms

Life is shaped by physics, and the biophysical dimension provides ecological sense to biochemistry [1]. Large bacterial multicellular aggregates can be conceptualized as physical entities, produced by “colonial organisms,” a term referring to the bacterial tendency to form close associations during and after reproduction, resulting in the formation of colonies that occupy physical spaces. However, the term “colonial organism” is equivocal, as it might suggest that colonies can be regarded as distinct organisms of a higher hierarchical level than their cellular components. That may suggest that both bacterial cells and colonial multicellular collectives can be considered biological individuals, defined as independent units of selection [2]. However, this perspective could be construed as an exaggeration of biologicism, thereby overlooking the physical ramifications of cellular life. Conversely, the physics of bacterial colonies also determines a microspace-derived phenotypic specialization, leading to a collaborative “division of labor” among the colony’s various cellular components [3]. The term “development process” applied to colony formation tends to strengthen the concept of the colonial organism, with a programmed sequence of formation. However, the question of whether the collective macrostructure and its spread on surfaces is derived from the physical forces resulting from the shape and density of bacterial cells along different growth and death phases remains open [4].

In this work, the physical organization of colonies is reviewed, considering their development and spread across different types of surfaces, ultimately resulting in physical entities that modify the environmental spaces. We use the term “bacterial colonial organism” to refer to bacteria capable of forming physically relevant multicellular colonies in exploitable environments and that functionally benefit from this collective organizational structure. Conventional three-dimensional colonies contain a population of cells that is 2–3 times greater than that of colonies grown in liquid media, where cells are larger and loosely interconnected [5]. Colony-derived benefits include stronger cell-to-cell collaboration in the acquisition, exploitation, and storage of nutrients; resistance to environmental or pharmaceutical stress; resistance to immunological responses and predation; and the fostering of cell release and dispersal [3,6]. In general, three distinct colony shapes can be identified: 3D-shaped colonies, conventional domed colonies with a low perimeter-to-height ratio or a high volume-to-surface ratio, and medium-diameter/high-ratio colonies. The latter may occur when their shape can be inscribed in a circle but has a star- or fingered profile. The colonies, which are predominantly 2D-shaped, flatter, film-like, are commonly referred to as biofilms. These biofilms have a high perimeter-to-height ratio or a low volume-to-surface ratio. In both cases, colonies are physical structures, and this review emphasizes their physical causes and consequences, an insufficiently considered aspect in colony literature. In the following sections, we examine the self-construction of colonies depending on the type of surfaces they colonize, the differences in the physical structure of 3D- and 2D-shaped colonies, the building up of complex colonies, the location in mucosal spaces, and the consequences of their physical occupancy, also mentioning inanimate human-constructed objects and structures.

## 2. The Origin of Order in Bacterial Colonies

Does the order exhibited by bacterial colonies stem from an encoded form of collective organization embedded within the bacterial genome, or is it just a passive outcome of the physics of individual cells? Mechanobiology of colony formation is undoubtedly a pivotal field in the study of multicellular organization [7]. Colony formation follows the Eden growth model, in which cellular clusters randomly associate and occupy empty spaces, resulting in radial growth [8,9]. This randomness produces a certain degree of order. The spontaneous formation of 3D-shaped colonies, characterized by the piling-up of inert particles, is driven by physical forces. For example, the formation of dry-sand piles exhibits a self-organized internal structure, with the development of an architectural-like distribution of particles known as arching because of its resemblance to Gothic arches [10]. In this case, the physical force shaping a colony-like structure is primarily gravity, possibly in combination with minor variations in the surface properties of the sand particles, which can result in local clusters. That phenomenon is not expected to occur with other, entirely homogeneous particles with minimal (tangent) interactions, such as the balls utilized in ball-bearing mechanisms.

In the context of bacterial colonies resulting from local bacterial multiplication, physical forces are likely related to the expansive force of growing particles, and also to cell size, cell surface appendages influencing cell-to-cell adhesion, the tendency to form chains or filaments, asymmetric cell division, autolytic events, or the secretion of exopolymers, including structural molecules such as lipopolysaccharides or teichoic acids. These, and likely other physical forces, mentioned below, can vary across bacterial lineages, giving rise to distinct colony types. The dynamics of these forces are contingent on the prevailing environmental conditions. Temperature, humidity, osmolarity, acidity, oxygen concentration, light, radiation, or contact with fluids—most importantly, those containing nutrients or harmful solutes—generate spatial gradients. Additionally, physical forces can also be generated by tribological effects of bacterial growth, including electric signals generated by intercellular friction and the function of ion channels [11].

These gradient flows, and their intersections, may influence the size, shape, and metabolic activity of the bacterial cell according to its spatial position within the colony, creating microcompartments and a microspace-derived phenotypic diversification. In certain cases, the physical position of bacterial cells within the colony might change. Such changes can result from bioconvecting flows, cellular motion, changes in the orientation of the division plane, or chemotactic responses [12].

## 3. Bacterial Colonial Formation on Surfaces

In 1881, Robert Koch, heavily influenced by Fannie Hesse, the wife of one of his assistants, Walther Hesse, developed solid culture media using agar as a solidifying agent for nutritive broth, in such a way providing a colonizable surface where colonies develop [13]. Since then, the intuitive image of a colony for a microbiologist is a domed or flat, semisolid structure of variable size with a circular perimeter that forms on a surface after inoculating an agar plate. The colony results from in situ growth, proportional to the nutrient composition. Distinct bacterial species frequently produce distinctive colonies, and even subspecific groups can be associated with specific, recognizable colony types. Colony formation on agar plates enables the isolation, clonal purification, and identification of the inoculated bacterial types.

Surfaces determine colony formation. The architecture of a colony resulting from the inoculation of bacterial particles depends on the type of surface it contacts. Bacterial cells can recognize the conditions of the surface to which they are attached, using mechanosensors, mostly envelope proteins such as sensor kinases, detectors of pH changes, mechanosensitive channels detecting changes in osmolarity, or pili and flagella to evaluate adhesive and cohesive forces [14]. Hydrogel-type culture media, as with conventional agar plates, have a solid–gas interface, but only a solid phase occurs in deep culture tubes when the inoculum is mixed with melted agar. In the last case, the binding surface is created by the bacterial growth, but that requires a particular stiffness of the agar. At agar concentrations below 0.4% (*w*/*v*), 3D-shaped colonies are not formed. Higher concentrations give rise to spherical or elongated lenticular colony shapes [15]. The surface of conventional agar plates can be humid or dry, which also determines the type of colony. This is also dependent on the type of organism, i.e., whether it is flagellated or not (see Section 4). The proportion of weight loss during the first hours of incubation is a good marker of surface humidity. The hardness of the hydrogel surface can be modified by varying the concentration of the solidifying agent (typically agar). The progressive reduction in this concentration results in different soft-agar surfaces, approaching the liquid phase, and the colony size and architecture vary accordingly. Also, liquids have surfaces supporting colony-like growth. Liquids have gas–liquid interphases, which determine liquid surfaces, and liquid–solid interphases, such as the wall of a culture flask, where the fluid and the solid contact through a curved fluid meniscus. The surface of liquids in contact with the air is, in fact, due to surface tension, which arises from the mutual attraction of liquid molecules at the interface with the gaseous phase, forming a stretched elastic membrane. This structured interphase can be related to shear surface viscosity [16]. Viscosity is a fluid’s internal molecular friction that results in resistance to flow. The type and expansion of colony produced by unicellular organisms depend on viscosity: at high viscosities, compact 3D colonies occur; when viscosity progressively decreases, colonies become more disaggregated, producing “fingers”, and finally break into fragments. In this process, metabolic activity in colonies growing on low-viscosity or liquid surfaces induces flows that influence the spatial expansion of the collective structure [17].

The interphase of a high-viscosity fluid with air provides a surface that facilitates bacterial adhesion and 3D-shaped colony formation, as well as resistance to desorption under shear stress induced by fluid flow [18]. Bacterial motility, however, may decrease surface viscosity, thereby modifying colony morphology [19]. Cell migration and edge expansion do not necessarily require flagellation: the metabolic activity of bacteria, such as fermentation, creates an osmotic gradient that influences fluid flows and propels bacterial cells in a process of “swashing” [20]. Also, the intensity of fluid flow seems to regulate the trade-off between adhesion and the bacterial multiplication rate [21]. In our view, the colonies within the hydrogel are also developing on self-made surfaces; therefore, that should also occur, perhaps with different dynamics, in the colonies growing on planar surfaces. It has been postulated that there is a fundamental difference in colony morphological dynamics based on the accessibility of nutrients when growth occurs on surfaces versus growing in the interior of a hydrogel. 

Within liquids, bacterial microcolonies can arise if bacteria use other bacterial surfaces to aggregate, as in the case of bacteria that remain linked to progenitors forming chains, as in *Streptococci*; bunches, as in *Staphylococcus*; cubic packages, as in *Sarcina*; cords, as in *Mycobacterium*; or rafts, as in *Listeria* or *Corynebacterium*. The surface of inert suspended microparticles in fluids offers an opportunity for the adhesion of bacterial cells forming aggregates, or granules, as in the microbioparticles, as the “marine snow” in marine, river, or lake water columns [22]. This view makes a different interpretation of the possibility of colony development without obvious surfaces [23]. In summary, all surfaces, both organic and abiotic, including hard, human-made surfaces that contact nutritional resources or energy sources, can serve as substrates for bacterial colony formation across organic (humans, animals, plants), marine, and industrial environments.

## 4. The Physical Structure of 3D-Shaped Colonies

Microbial particles behave differently when contacting solid or liquid surfaces, humid solid surfaces, or viscous layers. Three-dimensional-shaped colonies growing in the interior of semi-solid deep culture media and, probably, in mucous layers covering epithelia, differ markedly in shape. In all these cases, cellular growth gives rise to various collective physical structures. In hard hydrogels, as in conventional agar plates, growth typically produces colonies with a domed or curved profile and a limited perimeter, thereby limiting access to nutrients and leading to cellular death or shape-modified bacterial cells. Accordingly, the morphology of these colonies tends to be unstable over time [24]. Highly motile species, such as *Proteus mirabilis*, take advantage of the thin liquid moisture to swarm over a wide plate surface using hyperflagellated cells, resulting in a periodic ring pattern [25]. Eliminating moisture with ethanol prevents swarming, and *Proteus* forms regular colonies. Non-motile or low-efficiency motile bacteria growing on the surface of a nutrient-providing hard environment, such as a regular agar plate, produce colonies. Growth depends on Fick’s law of diffusion, so that nutrients flow from the surroundings towards the colony [26].

Mathematical models have been developed to reproduce the dynamics of colony formation using reaction–diffusion differential equations that account for growth or stress (even death) at different nutrient, water, and bacterial concentrations, as well as substrate softness [27,28]. These models are valid, except for the Eden-like mode of colony growth [8], in which new cells randomly attach to the perimeter layer. In all other cases, the process of growth toward the dome-structure of the colony leads to the emergence of slow-growing, frequently filamentous cells, as well as small, persistent cells. In both cases, these modified cellular shapes result from nutritional stress. In general, colony morphology depends on the coexistence of active and inactive bacterial cells [29], with active cells located in the colony periphery and inactive cells in the interior [30]. Only peripheral cells at the edge and, to a minor extent, on the surface can replicate efficiently. At these locations, the most successful genetic variants might grow, a process known as genetic demixing [30,31,32].

**Figure 1:**
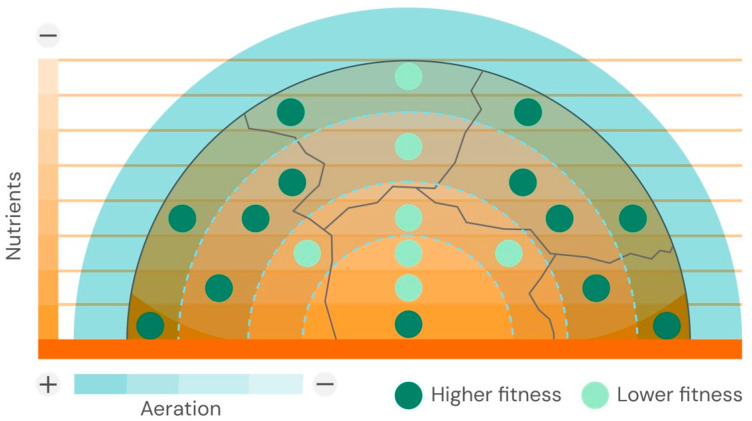
Schematic of compartments formed inside a 3D-shaped colony due to the intersection of different gradients. In the figure, gradients of aeration (in light blue) and nutrients (in orange) create local microecological heterogeneities, where bacterial cells (green dots) have varying levels of fitness, with lower fitness shown in light green.

In addition to nutrient gradients, physical constraints also contribute to colony formation. During this process of colony expansion, a verticalization of the interior cells occurs, driven by mechanical constraints, ultimately resulting in a slow-growing, nutritionally starved “death zone” [33]. Other types of cellular variation might alter the colony’s physical properties over time. Allolytic processes, such as cannibalism and fratricide, likely evolve, and cellular apoptotic death provides new nutrients to the survivors [34]. Water-nutrient capillary forces at the liquid–air interface in contact with particulate cell structures tend to fracture an otherwise homogeneous colony structure, creating densely packed cell clusters separated by bioconvecting flows [35]. Motile bacteria, such as *Salmonella*, appear to have an advantage in hydrating their colonies on culture plates, as flagella may help attract water to the agar surface [36]. However, it is worth noting that colonies of bacteria from different taxa exhibit different densities, with Gram-positive bacteria being harder and less hydrated, as shown by light-scattering diffraction pattern analysis [37]. A schematic representation of the internal structure of a 3D-shaped colony is shown in Figure 1.

## 5. The Physical Structure of 2D-Shaped Colonies, the Biofilms

Water constitutes 90% of the composition of a bacterial biofilm [38]. This fact constitutes a significant difference from hyperosmotic regular colonies growing on agar plates and may explain the unexpected observation that protein profiles of biofilms more closely resemble what is expected for exponentially growing cells in a liquid environment than those of cells reaching the stationary phase in colonies [39,40]. Water is retained primarily in the biofilm due to its exopolysaccharide composition. Adhesion to a surface facilitates the formation of local cellular microcolonies, which can sometimes grow large enough to resemble small mushrooms. The whole structure is determined by physical forces, such as cell-to-cell steric displacement of neighboring cells, bridging attraction mediated by electrostatic interactions, depletion attraction due to growth-dependent reduction in the exopolymer space, and osmotic pressure, which attracts fluids containing nutrients [7]. The expression of biofilm biomolecules follows a developmental process involving cellular signaling [41]. Such cell-to-cell communication involves quorum-sensing systems, which utilize autoinducers—chemical signals that modulate the expression of diverse genes in a population-density-dependent manner [42]. Mechanosensors that can recognize the nature of the colonized surface also act as a signaling system. Possibly, flagellated bacteria have specific surface recognition abilities, derived from physical signals originating from impediments to flagella rotation [43]. The origin of biofilm as an alternative lifestyle occurs in *Pseudomonas* or *Stenotrophomonas* following adhesion to surfaces and the activation of the chemoreceptor WspA, which leads to the phosphorylation of WspR, a regulator of cyclic-di-GMP synthesis. The effector protein in the cyclic-di-GMP metabolism is the Alg44 protein, a key member of the alginate operon leading the synthesis of alginate, the matrix exopolysaccharide where the cells are embedded [44,45].

**Figure 2:**
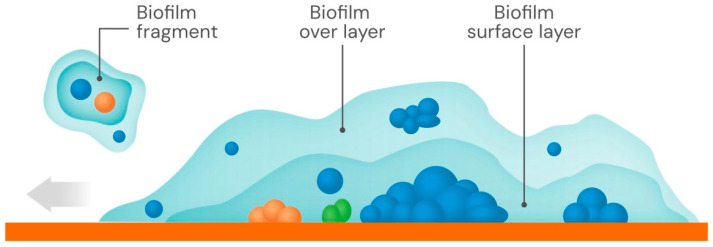
Schematic illustration of a biofilm, a predominant two-dimensional colony. The initial founder populations of the biofilm (blue spheres) on a surface produce an exopolymer (blue area). The horizontal spread of the exopolymer across the surface, caused by slippage of the overlayer (light blue), may capture preexisting colonies and microcolonies of other organisms (orange and green spheres), leading to a complex multispecies biofilm. Horizontal spread (grey arrow on the left) is facilitated by prior exopolysaccharide trails and is mediated by type IV pili and adhesins. Fragments of the biofilm may be detached (upper left), favoring colonization of other surfaces, including those of other biofilms.

As stated before, despite the highly dynamic and heterogeneous nature of biofilms, they tend to be much larger in extension and frequently thinner (predominantly 2D) than a domed colony (predominantly 3D) of the same species. This facilitates access to oxygen and increases access to nutrients in wider spaces. The hydration of the biofilm facilitates both nutrient flow and dispersion-mediated growth. A biofilm has a layered structure, with two separated main layers: the foundational layer attached to the surface, surmounted by a more mobile surface layer [46]. The upper layer may slide over the attached one, allowing efficient biofilm spreading [47]. Sliding probably depends both on repulsive interactions between cells and exopolymers and the size and density of the coat [48]. Both the primary adhesion events and the biofilm spread are limited by the local concentration of biosurfactants, molecules—such as rhamnolipids—capable of reducing surface tension at air/liquid/solid interfaces [49]. The stiffness of the surface contributes to the biofilm spreading rates [50]. Also, the chemical composition of the hydrogel substrate influences the biofilm–hydrogel interactive mechanics; as an example, the presence of tryptone in culture medium facilitates bacterial crosslinking of hydrogel polymer chains [51]. A schematic figure of the structure of a biofilm and its superimposed layers is presented in Figure 2.

There is a fuzzy zone between biofilms and mucoid colonies. *P. aeruginosa* colonies embedded in a dense alginate coat, *E. coli* variants hyperproducing colanic acid, or hypercapsulated strains of *Streptococcus pneumoniae* or *Klebsiella pneumoniae*, frequently retain the typical colony shape, but they look like shining biofilms, particularly following colony coalescence. Mucoid colonies are in fact composed of individual cells with a dense exopolymers coat, so that the biofilm is individual and also collective.

## 6. The Building of Complex Biofilm Architecture: Engulfment, Fusion, Co-Construction, and Invasion

On surfaces, whether biotic or abiotic, mainly comprising animal and plant tissues, as well as mineral, marine, and industrial environments [22], traditional bacterial colonies (mostly microcolonies in nature) can coexist with emerging or developing biofilms in the same space. These colonies could correspond to species different from those that formed the biofilm (polygenetic colonies). The shape of a multispecies colony depends on the presence and proportions of its component taxa, including minority populations [52]. Surface-displayed autotransporter proteins, and probably other specific surface components in each species, lead to auto- and hetero-aggregation [53]. The spatial interactions and dynamics between separate colonies and expanding biofilms have rarely been studied [54]. However, the spread of a biofilm across a surface can *engulf* resident colonies, covering and embedding them within the extracellular polymeric substance matrix, altering the local microecology for multiple partners. From the colony’s perspective, competition of different species for nutrients may be offset by increased nutrient flux and protection against harmful conditions, such as high osmolarity and unstable or turbulent flows. Fusion of nearby biofilms is expected to happen among biofilms of the exact clone, but possibly among related species as well. In some cases, it has been shown that biofilms are co-constructed through the cooperation of different species. For example, the presence of *Streptococcus mutans* can stimulate the up-regulation of genes involved in *Bacillus subtilis* extracellular polymeric substance production. Conversely, amyloid-like fibers produced by *B. subtilis* can enhance adhesion to *S. mutans* biofilms [55]. Cross-species communication may occur through universal quorum-sensing autoinducers [56]. Another possibility for building complex biofilms is the invasion of the biofilm by external bacteria. Apparently, this can be limited to motile bacteria, as immobile cells, even if clonally identical to those forming the biofilm, cannot penetrate the exopolymer coat [57].

## 7. Complex Biofilm Development in the Space, Organization, Maturation, and Reproduction

Biofilm formation resembles a multicellular development process [58]. Using 3D-morphometric analysis, the physical evolution of a biofilm has been compared with the process of urbanization [59]. The pioneer adherent settlers remain static but give rise to aggregates (villages), which then produce densely packed microcolonies (cities), which might merge, resulting in macro communities with spatially separated microbial types, some of which grow faster than others. That is the case for denser aggregates [60]. It is worth noting that the biofilm attachment surface is not necessarily homogeneous, either flat or curved, as in laboratory experiments. The physical, chemical, and biological heterogeneity of the colonized area’s natural topography determines the various merging combinations and the scaffolding structuration of bacterial aggregates. In multicellular biofilms, a complex network of interactions occurs, including beneficial interactions such as nutritional synergies, cross-quorum sensing, and 3D scaffolding, which result from bacterial filamentation. Biofilm expansion frequently follows the formation of cellular chains, which are favored in *E. coli* by a self-associating autotransporter protein concentrated at the cell poles [61]. Competitive interactions, including the release of antibacterial molecules, result in spatial segregation [62]. However, the final complexity of a biofilm is provided by niche diversification, allowing the emergence of physiologically distinct populations even within initially clonal populations [63]. Biofilms submitted to heterogeneous growth tend to be deformed; in combination with intra-biofilm fluid flow and shear stress, fragments of biofilm are detached from the surface [64] and can adhere to alternative surfaces in a process resembling biofilm reproduction and coalescent recombination with other biofilms (Figure 2).

## 8. Bacterial Colonies in Mucosal Spaces and Tissues

Natural body surfaces, including mucosal layers and tissues, have a spatially structured, irregularly porous architecture that allows fluids of different viscosities to flow over time with varying permeabilities. This environmental complexity creates a multiscale landscape shaped by numerous intersecting gradients of fluidity, nutrient availability and consumption, oxygen levels, or the presence of harmful substances. These factors lead to various chemotactic and quorum-sensing responses, influencing the build-up of different bacterial biomass, whose interactions also contribute to the habitat’s complexity [65]. In human mucosal environments, such as the gut or respiratory tract, this gradient heterogeneity and biomass buildup occur across spaces and surfaces of vastly different sizes, from microns (microvilli, ciliary structures) to millimeters (villi, crypts), centimeters (periodontal sacks, pharyngeal crypts), decimeters, or meters (respiratory tract branching, sections of the intestinal tract). Additionally, many of these structures are unstable, involving both vertical instability, as in the continuous shedding of cells resulting from mucosal turnover, and horizontal instability, as in specialized functions such as the respiratory ciliary escalator or intestinal peristalsis, which contribute to the spatial transmission of bacterial suspensions. The composition of mucosal biofilms depends on the host’s physiology, including variable nutrient availability, hydration of the mucin layer, the intensity of the innate immune response, and changes that occur over time or in pathological conditions. Mucosae are frequently covered by mucins, highly glycosylated and densely latticed proteins in a highly hydrated and viscous hydrogel. In addition, this gel contains antimicrobial peptides [66]. This antibacterial shield may promote the formation of a less protected, colonizable mucin surface where colonies can develop, thereby restricting mucin-layer invasion. In fact, an important portion of gut microbiota is attached to the mucin layer, forming complex biofilms [67]. In addition, the diversity of bacteria attached to mucosal surfaces differs from that of the intestinal lumen [68]. In the respiratory tract, complex biofilms are also formed, and such collective organization is critical in the pathogenesis of chronic infections [69,70].

During infection, when confined within tissues, bacterial microcolonies and larger colonies form, and their shapes depend on the shapes of the forming bacteria. Cocci-shaped bacteria tend to form compact, spherical colonies, and rod-shaped bacteria produce elongated colonies with higher access to nutrients [71]. In abscesses, as in those of *Staphylococcus aureus*, bacteria produce quasi-spherical aggregates or colonies confined by, in part, bacterial-induced deposits of fibrin cloths [72]. On hard, planar surfaces, such as dental surfaces, bone periosteum, cardiac valves, intravascularly colonized catheters, and other implanted foreign surfaces, the biofilm mode of growth predominates, including in cocci. If the attached bacteria are surrounded by flowing fluids, such as blood, 3D-shaped colony compact ensembles develop on hard surfaces, such as cardiac valves in endocarditis. In this case, the result is the formation of large protruding structures, the vegetations; essentially bacterial compact colonies inside a meshwork of fibrin [73]. Air-exposed skin lesions, such as burns, offer a nutrient-rich, hard but humid surface where bacterial biofilms develop [74]. The expansion of colonies in body tissues occurs by a combination of growth and active motility, giving rise to satellite colony formation, which facilitates a succession of colony coalescences and expansion of the population [75]. However, we cannot rule out passive motility linked to the flow of organic fluids and phagocyte migration at the periphery of the colony.

The physical structure of 2D- and 3D-shaped colonies in mucosae, within tissues, and on hard surfaces within the body ensures protection for the host’s immune response [76,77]. Because of the high bacterial density and the presence of slow-growing and persistent cells, colonies, even composed of susceptible bacteria, are poorly susceptible to antibiotic action. In addition, if the colony integrates resistant organisms, a minority of them, producing beta-lactamases, may protect the whole colony population from beta-lactam antibiotics [78].

## 9. Microecological Compartmentalization and Bacterial Colonies’ Physical Occupancy

**Figure 3:**
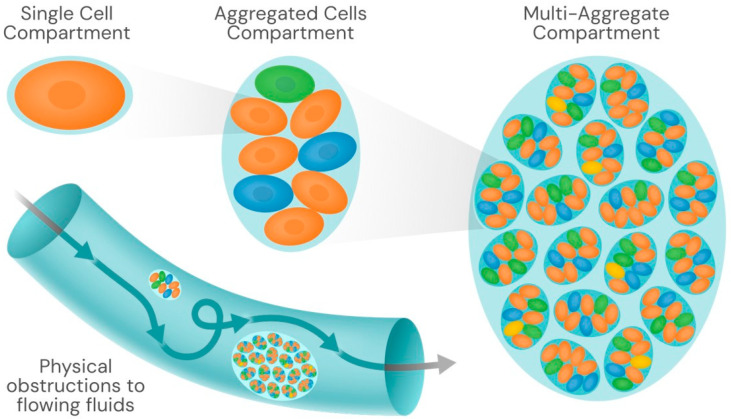
Bacterial cells, aggregated cells -which can be different- and multi-aggregates constitute physical compartments. Space occupancy by larger physical entities may influence the flow of fluids within the lumen of animal anatomical structures, from vascular or bronchial trees to intestinal or urinary tracts. They may also have microecological effects in the environment.

The term microecology is somewhat elusive and is generally used to describe ecological interactions between organisms at the microscale. In nature, bacteria are frequently located in confined spaces, which influences their collective behavior [79]. Colonies are compartments, and their habitat occupancy contributes to the physical structuration of the environment, influencing niche partitioning (Figure 3).

Clonal colonies, resulting from the same genetic lineage, are first-order physically confined compartments that prevent occupancy by alien organisms and ensure their own preservation. For instance, without the centripetal force of colony compartmentalization, autoinducers are washed out by free diffusion in liquid flows, thereby reducing the collective-action advantage. It has been proposed that the production of autoinducers is secondary to diffusion sensing or gradient sensing, thus identifying a compartmentalized landscape [80]. A colony is an exclusive, self-produced, stable niche-compartment for the colony-forming organism, as there is a fundamental organism-niche ontological correlation [81]. These compartments are certainly physical entities, ranging in size from hundreds of microns to millimeters or centimeters, and thus influencing the microenvironment independently of their biochemical activities, simply by occupying space, altering the microenvironment’s fluidity, and thereby interfering with the progression of physical and chemical gradients, including nutrient gradients.

A second-order physical compartment occurs when colonies of different species or variants of the same species share a common space, but are composed of several interconnected niches. This occurs when the components of the heterogeneous community in the resulting biomass gain an advantage from the collective physical entity, occupying more space, being more resistant to environmental stress, and facilitating trophic interactions. Such feeding interactions, determining nutrient flows, are highly dependent on physical compartmentalization [82]. Both intercolonial cooperation and antagonism facilitate compartmented coexistence in neighboring niches [83,84]. Under these spatially structured conditions, accelerated evolution of exploitative interaction between coexisting species is expected to occur [85]. Coexistence of related bacteria in microbiota is favored by niche partitioning derived from resource partitioning, allowing separate colony formation [86]. In the intestinal and, probably, the respiratory microbiota, host secretions from epithelia also contribute to niche partitioning [87]. The bacterial mass-driven, compartmentalized heterogeneity of the physical and biological microenvironments in the chronically colonized bronchi of cystic fibrosis patients probably explains the high diversity of *Pseudomonas aeruginosa* colonial morphotypes found in lung secretions, each occupying a different microecological compartment [88]. Host surface secretions may even confer greater fitness for co-colonization with different organisms in the case of symbiotic cooperation. The heterogeneity of host spaces contributes to the different patterns of coexistence, such as intestinal lumen versus crypts [89].

The third-order physical compartment is characterized by the formation of large, thick (centimeter-scale) microbial macroscopic mats, which are considered highly relevant in geobiological ecosystems. Microbial mats are a kind of mega-biofilm formed from the prior settlement of bacterial biofilms, integrating complex communities of microorganisms interacting with each other and their physical environment [90]. Mats generally have a multi-layered structure comprising both 2D- and 3D-shaped colonies and frequently constitute a self-sustaining ecosystem [91]. Microbial mats provide the biological and physical bases for the development of benthic complex communities involving bacterial, archaeal, protistan, microalgae, fungal, and invertebrate cooperative interactions, generally in marine-soil interphases. Such a mega-biofilm extends along 2,5 million km of continental coastline, making it the largest single ecosystem on Earth and providing critical ecosystem services [92,93]. The evolutionary importance of these mega-biofilms as hotspots for genetic interactions and as drivers of future evolution toward better-adapted life forms has been poorly explored. We know that stromatolites, the oldest fossils on Earth, were active bacterial mats that served as the cradle of early life evolution [94]. These mats were and are physical bioarchitectural constructions derived from the shapes and functions of microorganisms.

In summary, microbial colonial organisms are also physical entities that can exert occupancy effects, thereby modifying the local ecology and converting biological activities into physical hindrances. The consequences of physical changes imposed by colony formation under different ecological conditions are insufficiently known. In the more extensively explored field of human and animal health [95], the presence of bacterial colonies that adhere to the endothelial cell surfaces in organic cavities has been shown to result in deleterious effects. In the vascular system, colonies have been observed to obstruct capillaries and alter blood flow, even in large blood vessels, which is attributed to the colonies’ tendency to resist mechanical shear stress [96]. Additionally, the physical disturbances induced by colony-forming bacteria in the fluid flow within indwelling catheters, root canals, pharyngeal crypts, the osteocyte-lacuno-canalicular network inside cortical bone, milk ducts in the breast, or small-diameter bronchi are well-known examples of conditions that facilitate the recurrence of human infections. Certainly, similar micro-obstructions by bacterial colonies should occur in plant pathology, in marine environments [97], and also in the necrosis of stony corals.

There is a bacterial-colony-derived pathology affecting industrial constructions and devices, ranging from medical devices to architectural pieces to oil and gas pipelines and tanks. Biofouling, particularly in engineered structures that are frequently humid or submerged in water, occurs when microorganisms accumulate on inert materials, often leading to biofilm-driven corrosion (microbiologically influenced corrosion, MIC) [98]. The corrosion electrochemical processes produce material degradation (primarily metals), which creates novel physical microecological spaces where bacterial colonies might survive, as metals may adsorb nutrients [99]. On the other hand, biofilm-associated corrosion by itself impedes rapid liquid flow, which in turn favors colony formation [100]. Similar to the case of the physical effects of bacterial colonies in living tissues or in medical indwelling devices, anti-colony technology has been applied to inert surfaces, using biocidal coating or nanotechnology-based antifouling solutions [101]. The development of methods for colony detection in living tissues, including immunofluorescence methods or combined Mass and Surface-Enhanced Raman–Mass spectroscopy, might open new ways to target bacterial masses using laser-generated shockwaves [102]. In cases of colony formation on primarily abiotic surfaces, such as metal surfaces, techniques for evaluating biofouling [103] and for applying preventive or corrective interventions [104] may be useful. In summary, microbiologists have predominantly focused on the effects of chemical cues on bacterial behavior, but biophysics, the mechanics associated with biology, may offer a fertile perspective for future research in microbial ecology [14].

## 10. Conclusions

Bacterial reproduction on surfaces produces multicellular colonies of varying shapes and densities, enabling collective action within the underlying or peripheral spaces. Such action is both biological and physical. Although the biology of bacterial colonies has been extensively studied, the effects of colonies as physical structures occupying microecological spaces and altering fluid flow have received less attention in the literature. These effects are biophysical, meaning that biology and physics contribute to the colony’s effects on living organisms and on inanimate materials. Future trends in detecting colonies inside organisms or on industrial products, devices, and structures will enable targeted anti-colonial interventions, mitigating the deleterious consequences of these physical entities.

## Data Availability

No datasets were analyzed or generated during the study.
